# Child Health Service Nurses' Experiences of Providing Support to Parents With Overweight During Clinical Encounters About Their Child's Overweight—a Qualitative Study

**DOI:** 10.1111/cch.70200

**Published:** 2025-12-08

**Authors:** Julia Lindblom, Elzana Odzakovic, Eric A. Hodges, Anne Christenson, Maria Björk

**Affiliations:** ^1^ Department of Nursing, School of Health and Welfare Jönköping University Jönköping Sweden; ^2^ CHILD Research Group Jönköping University Jönköping Sweden; ^3^ School of Nursing The University of North Carolina at Chapel Hill Chapel Hill North Carolina USA; ^4^ Center for Obesity, Academic Specialist Center Stockholm Sweden; ^5^ Department of Medicine Huddinge Karolinska Institutet Stockholm Sweden

**Keywords:** family support, health personnel, obesity, paediatric, parent, qualitative research methods

## Abstract

**Background:**

Childhood overweight poses health risks and often persists in adulthood, making early intervention essential. Swedish Child Health Service (CHS) nurses are responsible for addressing this issue, a task perceived as important yet challenging. When supporting these families, a caring approach is crucial for well‐being and adherence. Childhood overweight is often associated with parental overweight. Due to the sensitivity and stigma surrounding adult overweight, healthcare professionals may experience discomfort, potentially leading to negatively perceived interactions. Despite these challenges, little is known about how parental overweight influences CHS nurses' approach in encounters regarding children's overweight. Therefore, this study aimed to explore how CHS nurses experience providing support to parents with overweight during clinical encounters about their child's overweight.

**Methods:**

Qualitative descriptive, inductive design was applied. Individual semi‐structured digital interviews were conducted with 14 CHS nurses, and transcripts were analysed using latent content analysis.

**Results:**

Four categories were identified: feeling insecurity regarding professional role, focusing on concrete aspects of health, questioning the parent and trying to influence the parent. CHS nurses perceived addressing the child's overweight as their primary duty. They focused on weight‐related aspects of the child's health, emphasising lifestyle advice, while parents' emotions or socioeconomic challenges risked being overlooked. Parental overweight could trigger negative assumptions, including doubts about parents' ability to support their child. When children's weight curve remained unchanged, frustration sometimes led CHS nurses to emphasise parental responsibility or use threats, such as involving child welfare services.

**Conclusion:**

This study highlights how CHS nurses perceive both challenges and responsibility in addressing childhood overweight, difficulties that intensify when parents themselves have overweight. The findings suggest that while striving to fulfil their role and balance professional responsibility with maintaining a supportive and caring approach, CHS nurses' are sometimes shaped by weight stigma.

## Summary

- CHS nurses face challenges in clinical encounters about a child's overweight when the parent also have overweight. They are driven by a strong sense of professional responsibility, which seems to be complicated by the parents' overweight.
- CHS nurses may adopt an approach that is influenced by societal weight stigma, which can lead to distrust of parents and strained interactions.
- More guidance is needed to support CHS nurses in complex conversations about a child's overweight when the parent also has overweight.
- Future research should explore how CHS nurses construct their professional role and how they navigate—and potentially reinforce or resist—weight stigma in clinical encounters.

## 1 Introduction

Overweight among adults and children has increased in the last few decades, tripling worldwide since 1975 (Abarca‐Gómez et al. 2017; World Health Organization 2025). In Sweden, trends in overweight have followed a similar pattern (Folkhälsomyndigheten 2025a; Folkhälsomyndigheten 2025b). Due to its associated health risks (Twig et al. 2016; Chen et al. 2024), the World Health Organisation (WHO) (2025) emphasises the importance of early prevention and management, placing responsibility on the healthcare system. In Sweden, the National Board of Health and Welfare (Socialstyrelsen 2014) states that the Child Health Service (CHS) plays a crucial role in early interventions to address childhood overweight, in close collaboration with the parents.

Childhood overweight is often associated with at least one parent having overweight (Alberga et al. 2019). Studies focusing solely on childhood overweight show that CHS nurses view this work as an important duty (Nygaard and Øen 2024), yet they sometimes feel uncertain about how to address the topic with the parents, which can lead to frustration (Castor et al. 2021). CH nurses also regard building a caring relationship with parents as essential, while worrying that raising this sensitive topic could jeopardise that relationship (Sjunnestrand et al. 2019). Research focusing on adults with overweight similarly shows that healthcare personnel perceive these encounters as complex and demanding and that the sensitivity of the topic can make it challenging to raise the issue (Paine et al. 2023; Bräutigam Ewe et al. 2021).

Adding to this complexity, healthcare personnel may hold weight stigma—defined as prejudiced or discriminatory attitudes and behaviours (Puhl and Heuer 2010)—towards both adults with overweight (Lawrence et al. 2021) and parents of children with overweight (Gothilander and Johansson 2023; Halvorson et al. 2019). Experiencing such stigma affects not only patients' health (Westbury et al. 2023) but also their trust in healthcare professionals, and may lead patients to adopt a defensive stance in future healthcare contacts (Paine et al. 2023; Wetzel and Himmelstein 2024). Earlier research found that parents of children with overweight who experience such stigma may feel fear, shame and powerlessness and may dread or avoid making appointments for their child (Gorlick et al. 2021; Haqq et al. 2021).

CHS nurses' health‐promoting work includes supporting families regarding children's overweight in a caring and non‐stigmatising manner to enhance the quality of care (Socialstyrelsen 2014; World Health Organization 2018). Caring, defined by Swanson (1991, p 165) as ‘a nurturing way of relating to a valued other towards whom one feels a personal sense of commitment and responsibility’, is also central to patients' health outcomes (Hogg et al. 2018). In the CHS context, research shows that CHS nurses' caring behaviour in conversations about childhood overweight can either empower parents or, if perceived negatively, undermine their trust and willingness to follow advice (Fridolfsson et al. 2024; Eli et al. 2022). Previous research shows that building a caring relationship with both parents of a child with overweight (Sjunnestrand et al. 2019) and adults with overweight (Paine et al. 2023) is considered an important yet challenging task. Studies have also demonstrated that health care personnel may direct weight stigma towards both these groups (Lawrence et al. 2021; Halvorson et al. 2019). However, little is known about how CHS nurses experience encounters when both the parent and the child have overweight.

Therefore, the aim of this study was to explore how CHS nurses experience providing support to parents with overweight during clinical encounters about their child's overweight.

## 2 Method

### 2.1 Design

This study used a qualitative descriptive design (Patton 2015), employing an inductive approach (Elo and Kyngäs 2008). This study adheres to the Standards for Reporting Qualitative Research (SRQR) to ensure transparent and complete reporting of the qualitative research process (O'Brien et al. 2014).

### 2.2 Context

The Swedish CHS provides free services to all children aged 0–5 years, with an attendance rate of 98%–99% (Svenska Barnhälsoregistret 2024). The visits are primarily conducted by nurses specialised in children's health, with physicians present at four of the 17 scheduled health visits. Additional interventions beyond regular visits can be implemented when needed (Rikshandboken Barnhälsovård 2024). CHS nurses are required to provide support and collaborate with parents, focusing on health promotion, growth monitoring and preventive care. This includes early detection and management of childhood overweight through structured, individualised lifestyle support with parents (Socialstyrelsen 2014; Socialstyrelsen 2024).

### 2.3 Participants

CHS nurses were recruited using purposive sampling (Polit and Beck 2016). Inclusion criteria were (1) having an education as a public health nurse or paediatric nurse, (2) working at a Child Health Service Centre (CHSC) in Sweden, (3) being verbally fluent in Swedish, and (4) having experience in providing support to parents with overweight during clinical encounters about their child's overweight.

Participants were recruited via social media platforms targeting nurses (e.g., the Facebook groups ‘The Nurse’), public health nurses (e.g., ‘The Swedish Association of District Nurses’) and CHS nurses (e.g., ‘CHS Nurses in Sweden’). Author JL contacted the administrators of these groups to obtain permission to post the invitation. Once approval was granted, a standardized recruitment message containing study information and contact details was posted.

In addition, CHS managers from 11 of Sweden's 22 regions granted permission to distribute study information to their nurses. The remaining regions either declined or could not be reached. The participants who ultimately agreed to be interviewed came from six of these regions, representing northern, central and southern Sweden.

In total, 19 nurses expressed interest in the study. Three withdrew due to high workload and two failed to respond after initial contact. This resulted in 14 CHS nurses participating in the study. The nurses' characteristics are presented in Table 1.

**TABLE 1:** Characteristics of participating CHS nurses.

Characteristics	Participants, *n* = 14
Age, mean (range)	44 (29–53)
Gender, *n* (%)	
Female	14 (100)
Education, *n* (%)	
Paediatric nurse	7 (50)
Public health nurse, n (%)	7 (50)
Years as a specialist nurse, *mean (range)*	9 (1–19)
Years at CHSC, *mean (range)*	8 (1–19)
Education targeted at childhood overweight, *n* (%)	4 (29)
Geographic representation, *n* (%)	
Northern Sweden	2 (14)
Mid‐Sweden	3 (21)
Southern Sweden	9 (64)
Work location, *n* (%)	
Larger city (> 50 000)	8 (57)
Smaller city (> 15 000)	0 (0)
Rural municipality (< 15 000)	6 (43)

### 2.4 Data Collection

Data were collected through semi‐structured interviews, to enable in‐depth exploration of the participants' experiences (Patton 2015). The interview guide was developed by the authors JL, EO and MB, drawing on existing literature and the study's research aim. Prior to data collection, the interview questions were pilot tested by author JL with two CHS nurses, and refinements were made based on their feedback. The interviews were conducted by author JL, between October 2021 and March 2022. The sample size was guided by the concept of ‘information power’, which suggests that the more relevant information the sample holds for the study aim, the fewer participants are required (Malterud et al. 2016). Based on this principle, the sample was considered adequate when no further information was discerned. The interviews were conducted via video using the software programs Zoom or Microsoft Teams, depending on the participants´ preferences.

After collecting demographic data about the participants, the interviews began with an open‐ended question: ‘Tell me about your experiences of supporting parents with overweight when they visit CHS regarding their child's overweight’ to encourage CHS nurses to discuss factors they associated with the topic. Probing questions, such as ‘Can you tell me how you gained an overall picture of this family's situation?’ and ‘What were your thoughts at that time?’ were asked when needed to delve deeper and clarify any ambiguities.

The interviews ranged in length between 54 and 98 min (mean 73 min), and each participant was interviewed once. All interviews were audio recorded and transcribed verbatim by either author JL or a hired transcriptionist.

### 2.5 Data Analysis

Content analysis is a systematic way of exploring and understanding phenomena by identifying connections and patterns in large amounts of material, such as interviews (Elo and Kyngäs 2008; Vaismoradi et al. 2013). In this study, qualitative latent content analysis following the steps recommended by Elo and Kyngäs (2008) was employed.

The analysis started with the *preparation phase*, where the transcribed interviews were read several times to create a sense of the data and gain an understanding of participants' experiences as described in the data. Author JL then highlighted sections that represented the participants' experiences of the phenomenon, which served as the units of analysis. During the *organising phase*, codes shedding light on different aspects of the highlighted sections were written in the margins. From these codes, subcategories were created freely to describe all aspects of the content. Based on their content, these subcategories were then grouped together into generic categories. Each subcategory and generic category was named based on interpretations of its content. This process was iterative, involving writing and re‐writing of the text, and grouping and regrouping of the codes and subcategories. For an example of the analysis process, see Table 2.

**TABLE 2:** Example of analysis process.

Meaning unit	Code	Sub‐category	Generic category
Is this going to be a bad conversation? Are they going to take it personally? If so, we will not be able to work with the child.	Parental reactions can hinder the work	To be unsure how to balance professional responsibility	Insecurity regarding professional role
I said, ‘Look at the curves here. That was the sum that came up. This is not something any of us made up; this is reality.’	The weight curve shows parents the reality	To focus on the measurable	Focusing on the concrete aspects of health
This is the case with parents who have struggled with their weight. They have such good knowledge and know exactly what I want to hear.	Parents know what you want to hear	To distrust parental descriptions of family lifestyle	Questioning the parent
To listen to them, support them as much as possible, and show understanding	To give support by listening	To show compassion	Trying to influence the parent

### 2.6 Ethical Considerations

Ethical approval was obtained from the Research Ethics Committee in Sweden, Dnr 2022‐03911‐02. This study followed the Helsinki Declaration (World Medical Association 2013). All participants received oral and written information about the study and provided written informed consent. To keep the participants' identities confidential, each was given a fictional name for the quotes.

## 3 Result

Four generic categories were identified: (1) feeling insecurity regarding professional role, (2) focusing on the concrete aspects of health, (3) questioning the parent and (4) trying to influence the parent. Each category had two related subcategories (Table 3). In the “Result” section, parents with overweight who have a child with overweight are referred to as ‘parents’.

**TABLE 3:** Overview of generic categories and related subcategories.

Generic categories	Subcategories
Feeling insecurity regarding professional role	To be unsure how to balance professional responsibility
	To be unsure about one's professional knowledge
Focusing on the concrete aspects of health	To focus on the measurable
	To focus on giving advice
Questioning the parent	To distrust parental descriptions of family lifestyle
	To distrust parental ability
Trying to influence the parent	To show compassion
	To wake the parent up

### 3.1 Feeling Insecurity Regarding Professional Role

This category describes how the CHS nurses strived to maintain their relationship with the parents while also advocating for the child's health, sometimes creating a difficult balance in their professional role. Parental overweight also appeared to make conversations about children's overweight more challenging, leading CHS nurses to question whether their knowledge of the topic was sufficient.

#### 3.1.1 To Be Unsure How to Balance Professional Responsibilities

Striving to maintain their relationship with these parents, CHS nurses approached discussions about the child's weight cautiously. Recognising that the societal stigma surrounding overweight could heighten the sensitivity of the topic when parents also had overweight, they aimed to avoid negative parental reactions.

However, as advocates for the child's health, the CHS nurses felt obligated to address the overweight issue, to achieve a plateau in the child's weight curve. Failure to do so was seen as malpractice, and a cautious approach towards the parents made the CHS nurses question whether they were neglecting their responsibility. Believing that parental overweight is a manifestation of unhealthy lifestyle choices further strengthened the nurses' sense of professional responsibility towards the child. This created a dilemma, as CHS nurses had to balance their responsibility to the child against the risk of straining their relationship with the parents. Sofia stated:

Some can become very offended, and that's difficult, but I still try not to avoid the topic (of the child's overweight) because I think it's important. We also have our role and our responsibility to talk about this.

#### 3.1.2 To Be Unsure of One's Professional Knowledge

As the CHS nurses perceived that parental overweight made conversations about childhood overweight more sensitive, they felt uncertain whether their knowledge of how to approach and support these parents was sufficient. Their belief that parental overweight reflected an unhealthy lifestyle also made these encounters feel more challenging compared with those where only the child was overweight. Therese described this as:

I think there might be some healthy habits in those families (where only the child is overweight)… that small changes are needed, but if you have parents with overweight, who have been overweight their whole lives maybe… there's a lot of heredity in this and habits, it's harder.

When their advice or support did not yield the desired results, the CHS nurses' insecurity increased further. However, longer experience within CHS fostered a sense of increased knowledge and self‐confidence, allowing the nurses to address the child's overweight more firmly. When unsure about their knowledge, the CHS nurses valued the possibility of referring families to other personnel, such as dietitians and/or psychologists. However, when referrals were not possible due to parental reluctance or limited resources, the CHS nurses felt they were alone and had to rely on their own knowledge. Tove expressed her uncertainty, stating:

I don't really know how to approach them. If I knew more, (…) then I wouldn't be afraid to talk about it (the child's overweight) with the parents in the same way.

### 3.2 Focusing on Concrete Aspects of Health

This category describes how the CHS nurses focused on measurable factors related to the child's health. While reluctant to address emotional topics, they emphasised the importance of providing concrete lifestyle advice, sometimes regardless of parental receptiveness.

#### 3.2.1 To Focus on the Measurable

The CHS nurses emphasised the importance of gathering measurable information about the child's daily life to get a picture of the child's health. This included eating habits, physical activity and the family's social context. To illustrate weight changes and facilitate discussions about lifestyle changes, the CHS nurses highlighted the weight curve as a crucial tool. They perceived it as an objective and concrete reality, particularly useful when weight was a sensitive issue or met with parental reluctance. Ida explained.

This mother probably hadn't really wanted to see it (the child's overweight), but it became so clear with this curve. So, she had probably hoped it wasn't as bad as it was (…) But now, it wasn't good at all. Now, there had really been a significant deterioration.

While some CHS nurses initiated discussions about parental overweight, others described shifting these conversations towards more measurable topics, such as setting goals for diet or physical activity. This approach aimed to avoid evoking negative parental reactions. There was also hesitancy in exploring parents' emotions, such as feelings of isolation in parenthood, as the CHS nurses felt ill‐equipped to handle these conversations. Tove expressed this as:

But I can talk about health… I mean, dietary habits and that part I can talk about, but not about the person per se, I mean not just about the parents themselves.

#### 3.2.2 To Focus on Giving Advice

Providing advice was a crucial aspect of the CHS nurses' approach when addressing childhood overweight. When striving to tailor their advice to each family, the CHS nurses emphasised collaboration with the parents. However, advice was also given based on established guidelines or personal preferences. It included practical suggestions, such as preparing homemade ketchup or squeezing fresh orange juice, even for families with financial or mental health difficulties. Stina said:

I think I take a leading role… I provide ready‐made solutions, 'Don't do that, do this, stop buying'… I take quite an active and aggressive role in it all.

While giving advice, the CHS nurses paid attention to parents' reactions, such as tone of voice and body posture, to identify the most effective approach for promoting lifestyle changes. These observations also helped them recognise when advice might not be well received. Despite this, the CHS nurses sometimes persisted in offering advice and sought ways to encourage reluctant parents to return for follow‐up meetings, such as scheduling routine check‐ups for younger siblings, based on the belief that such persistence could help parents make healthier choices.

### 3.3 Questioning the Parent

This category describes how CHS nurses questioned the trustworthiness and abilities of parents with overweight. Parental reactions and adherence to advice during clinical conversations about childhood overweight further influenced these perceptions.

#### 3.3.1 To Distrust Parental Descriptions of Family Lifestyle

The CHS nurses perceived that they could not always trust these parents' lifestyle descriptions, especially when they observed a discrepancy between the reported lifestyle, the child's weight curve and the physical appearance of both parent and child. They sometimes suspected that these parents might be deliberately untruthful, attributing this to a reluctance to reveal their own unhealthy habits. Jessica described her thoughts this way.

But then he (the father) says things that just can't be true. The child eats normal portions, no candy, and only lots of vegetables; it can't be true when it's… I mean, on paper here, we can see that there's something… and then the rest of the family too.

Due to the parent's overweight, the CHS nurses believed they had probably tried to lose weight themselves. This could lead them to believe that the parents understood what was expected of them and might agree to anything to present themselves positively or avoid scrutiny. This perceived lack of truthfulness was experienced as both provocative and tiresome. Kristin stated.

Unfortunately, this is how it is with parents who've struggled with their weight their whole life and lost, they have such good knowledge, they know exactly what I want to hear. So, it's really hard to get anything useful from them.

#### 3.3.2 To Distrust Parental Ability

The CHS nurses could express distrust in these parents' ability to promote a plateau in their child's weight curve, as they perceived the parents' overweight as a sign of weakness and a failure to manage their lifestyle. Despite this, the CHS nurses acknowledged that factors such as socioeconomic disadvantages and mental health challenges could impact parents' ability to implement lifestyle changes. Moreover, the CHS nurses expressed enhanced trust in the parents' ability to support their child when the parents demonstrated a willingness to implement changes suggested by the CHS nurses.

However, trust was undermined when parents became upset or uncomfortable during discussions about their child's weight. Such reactions could prompt the CHS nurses to question the parents' ability to prioritise their child's needs, as described by Anna.

You notice that they fidget and don't really want to listen … And then I feel like there is no one home … They have no concern about it, and then you feel (…), it's still for the child's best interests, you know.

In the absence of progress in the child's weight curve, the CHS nurses' trust in the parents' abilities to support their child was further diminished, sometimes leading to concerns about potential child neglect. Ingrid described this as:

It's frustrating, it becomes a bit sad too… because they were overweight and… they also passed it on to their children. (…) It is a form of abuse… and if you just think about it… it's almost grounds for a child welfare report.

### 3.4 Trying to Influence the Parent

This category describes how CHS nurses attempted to influence these parents to achieve changes in the child's weight curve. They sought to build trust and avoid blame. However, when parents appeared uninterested or when the child's weight curve remained unchanged, the nurses experienced frustration, which led them to emphasise the parents' role in implementing necessary lifestyle changes to support their child's health.

#### 3.4.1 To Show Compassion

By showing compassion, the CHS nurses aimed to support parents in promoting a plateau in their child's weight curve. They also sought to strengthen the parents' confidence in their ability to do so, believing that parents with overweight might have a greater need for such support. Lisa described this as.

Confirm so that she feels she's doing the right thing, I think it's really important for the parent (with overweight) to get that confirmation that you're doing the right thing, you're doing everything you can for your child.

The CHS nurses also tried to adopt a gentle and collaborative approach, using body language, active listening, additional visits, and sharing personal experiences of being overweight. Jessica explained.

To be gentle, try to show that I don't mean any harm, that I was there if she needed help and support … It's mainly about tone of voice, body language, and then the kind of questions you ask.

As part of this compassionate approach, and given the parents overweight, the CHS nurses tried to avoid personal blame. They avoided stigmatising words such as ‘fat’ or ‘overweight’ and instead discussed weight trends and the relationship between weight and height. Furthermore, they used heredity as an explanatory model, referring to the child's overweight as an ‘allergy to calories.’ This allowed them to avoid blame while making sure these parents understood the need to make more active lifestyle efforts than others. Kristin phrased this perspective as:

One has inherited certain genetics, and one must deal with that. You must see it as, unfortunately, she's almost allergic to calories… Just so it doesn't become so blameful, that unfortunately she has drawn the short straw… and that's really sad, but now we have to try to help her (the child).

#### 3.4.2 To Wake the Parent Up

When the child's weight curve failed to plateau despite the given advice, CHS nurses experienced frustration and powerlessness. While this lack of change could initially be perceived as a professional failure and evoke feelings of shame, over time, it could be reframed as a result of parental responsibility and attributed to perceived defensiveness or a lack of interest or motivation. This created a desire to wake the parents up to the seriousness of their child's overweight. To achieve this, the CHS nurses emphasised parental responsibility for their child's health and highlighted the risks associated with overweight. This was conveyed by mentioning that the child might experience bullying due to their weight or that children could later hold their parents accountable for allowing them to develop overweight. Stina stated.

I think it's important to … sell two messages … the child will suffer if we don't do anything. Suffer physically, mentally … their whole life will be … ruined in some way. And the other is … the message that we can do something about it.

While CHS nurses acknowledged the importance of respecting parents' wishes, they sometimes resorted to threats, such as making a child welfare report, to attempt to wake parents up and pressure them into following given recommendations. Anki described this as:

They knew … that I wanted to refer them to the obesity team … and I was pretty clear that if they don't accept … because the parents have the right to refuse … then I said, ‘Well, then I'm actually worried about the child.’ And, implicitly, they understood that … this could be a reason for me to file a child welfare report.

## 4 Discussion

In this study, we explored CHS nurses' experiences of providing support to parents with overweight during clinical encounters about their child's overweight. CHS nurses perceived addressing children's overweight as a central responsibility, and they prioritised weight‐related health and lifestyle advice. This focus could overshadow parental emotions or socioeconomic challenges. Parental overweight prompted negative and stigmatising assumptions and actions, including distrust in parents' accounts and caregiving ability. Lack of progress in the child's weight could lead to frustration, with nurses emphasising parental responsibility or making threats.

Uncertainty about the professional role was prominent in this study, but, as in previous research (Mäenpää and Vuori 2021; Harder et al. 2019), the CHS nurses considered addressing childhood overweight as their primary responsibility, even when parents were reluctant to do so. Our findings also suggest that parental overweight intensified this sense of responsibility. This approach may reflect society's weight‐centred focus (Rubino et al. 2020), where healthcare personnel are encouraged in policy documents to address overweight in all patient encounters (Region Stockholm 2024), are the target of interventions promoting such discussions (Goodfellow et al. 2016; Pallan et al. 2024) and are criticised when failing to do so (Warren and Hunt 2017). This approach also appears to be shaped by stigmatising beliefs framing weight as a choice, manageable by diet and exercise and the view that increased pressure leads to greater weight loss (Rubino et al. 2020; Turner 2024). Swanson (1993) describes a caring approach as involving respect for the other's free will and capacity to make their own choices, while acknowledging that the possibilities available are not always equally accessible, acceptable or desirable for everyone (Swanson 1993). Providing advice despite parents' expressed wishes disregards this autonomy and may have unintended consequences, as parents repeatedly subjected to unwanted weight conversations feel helpless and defensive (Eli et al. 2014), and unsolicited advice is less likely to be followed (Van Swol et al. 2017). This suggests that it is important to support CHS nurses to try to get to know the parents and families they meet—understanding their circumstances, needs and wishes (Swanson 1993) while also fulfilling their mission.

The CHS nurses in this study focused on concrete aspects of health and felt ill‐equipped to address parents' emotional reactions, steering conversations towards lifestyle advice. This may reflect a belief that their professional role is to advise parents in these specific areas, as described by Sela et al. (2022), or may result from a perceived lack of training in addressing parents' emotional responses, as shown by Nygaard and Øen (2024). Previous research also points out how healthcare personnel adopt a body‐focused approach, while social and psychological aspects are overlooked (Backman et al. 2020). This approach risks disregarding the broader determinants of health, as outlined by WHO (1948), as well as oversimplifying the complex causes of overweight. The aetiology of overweight includes genetic and hormonal factors (Michałowska et al. 2021; Willyard 2014), social determinants such as stress and financial strain (Hernandez et al. 2017; Cuevas et al. 2019) and environmental factors, including food‐environment and access to open space (Beynon et al. 2021). Furthermore, focusing on lifestyle changes and weight as the primary health outcome risks reinforcing weight stigma, as argued by Tomiyama et al. (2018). The measurable approach demonstrated in this study indicates that CHS nurses could benefit from a more holistic approach to health, as suggested by the EU's Health Development Model (Bauer et al. 2006) and Swanson (1993), who, from a caring perspective, describes health as being engaged in one's life, and emphasises the need for comprehensive support.

This study showed that CHS nurses questioned these parents' abilities and seemed to associate their overweight with weakness and failure. Similar views have been documented towards parents whose child is overweight (Gothilander and Johansson 2023; Isma et al. 2012; van der Voorn et al. 2023) and towards adults with overweight (Rubino et al. 2020; Palad et al. 2019), which may explain why parental overweight in this study appeared to trigger stigmatising thoughts and actions. Although this study does not explore parents' experiences, previous research has shown that stigma can contribute to elevated stress, higher BMI and increased mortality (Westbury et al. 2023; Puhl et al. 2020). Furthermore, a caring approach is crucial to the patient–health care personnel relationship (Turkel et al. 2018). One cornerstone of caring is maintaining belief in the other, while acting respectfully and nonjudgmentally (Swanson 1993). This contrasts with the CHS nurses' views and actions described in our study, where attempts to coerce parents into following advice risks reinforcing stigma and hinder the supportive and collaborative relationship on which good care depends (Watson 2012).

While the CHS nurses' attitudes displayed in this study are concerning, they do not exist in isolation but rather reflect broader societal norms (Rubino et al. 2020). Stigma is driven at social and socio‐ecological levels, as well as at the individual level, where a lack of awareness about weight stigma can perpetuate and reinforce it (Stangl et al. 2019). Paediatric healthcare personnel with weight bias are often aware of their biases and willing to address them. This prior research suggests that increasing awareness and providing structured support, such as anti‐weight bias education, may help CHS nurses resist these societal pressures and adopt a more supportive approach that aligns with maintaining belief in parents' abilities (Turner 2024).

The CHS nurses in this study sought to influence parents to support a healthier weight trajectory in the child. When this was not achieved, they could feel shame for failing in their professional responsibility, which was sometimes redirected as blame towards parents perceived as unmotivated or defensive. This aligns with previous research showing that perceived parental disinterest or lack of weight improvement can cause frustration among health care personnel (Gothilander and Johansson 2023) and that professional shame can trigger defensiveness and blame (Gibson 2014). However, parents may worry that discussing their child's weight could lead to future eating disorders (Eli et al. 2022; Kovacs et al. 2018), and parents with overweight may have encountered stigma in previous healthcare interactions (Puhl and Heuer 2010). Such experiences may help explain why the CHS nurses in our study perceived parents with overweight as defensive or unmotivated in conversations about their child's weight. To address these challenges, Swanson (1991) describes how the caring encounter should aim to understand parents' experiences without preconceived assumptions. By recognising the significance of others' reactions within their own lived reality, a meaningful path forward can be facilitated. By adopting this perspective, CHS nurses might approach parental reactions—such as distress or possibly withholding information —with a genuine desire to understand and move beyond their own assumptions. By reflecting on how they can provide a safe space for parents, they may also foster a meaningful path forward that considers all dimensions of health.

### 4.1 Strength and Limitations

The authors acknowledge the study's strengths and limitations. One limitation to consider is that the CHS nurses who volunteered may have had a stronger interest in the topic, and it is possible that other perspectives might not have been heard. Another limitation is the use of digital interviews, due to the possibility of technical issues and the challenges participants may face in establishing rapport (Thunberg and Arnell 2022). However, in this study, the participants were actively engaged in the interviews and rich, detailed and varied data were provided, thereby enhancing credibility (Lincoln and Guba 1985). The concepts and expressions identified in the study are also consistent with existing research and clinical experience within the research group, further reinforcing the credibility of the findings (Lincoln and Guba 1985). One strength of the study is the detailed description of the analytic process, and the use of quotations, that show that the interpretations were grounded in the data, thereby enhancing the confirmability (Lincoln and Guba 1985). Another strength of this study lies in the authors' collaborative approach during the data analysis process. Drawing on diverse clinical and research backgrounds, the authors engaged in open dialogue by comparing and discussing interpretations and insights related to the phenomenon under study, which strengthened the dependability. While author JL had overall responsibility for the analysis, regular discussions and systematic reviews of the coding and categorisation were conducted among all authors to ensure consensus, thereby enhancing the dependability of the findings (Lincoln and Guba 1985). Although a limitation of this study is that all participants were women and that only six of Sweden's 22 regions were represented, with the southern part somewhat overrepresented, this reflects the predominantly female composition of the nursing workforce (Socialstyrelsen 2021) as well as the population distribution in Sweden (Statistics Sweden 2025). This may therefore be considered representative of the professional and national context. The limited regional representation was largely due to challenges related to the COVID‐19 pandemic and high workloads among CHS nurses and managers. Nevertheless, these six regions are geographically distributed across northern, central, and southern Sweden and include areas with a high prevalence of overweight (Folkhälsomyndigheten 2025b), supporting the relevance and transferability of the findings. Moreover, the participants had varying work experience and levels of exposure to families dealing with overweight, which enriched the perspectives shared and enhanced the transferability of the findings (Lincoln and Guba 1985).

## 5 Conclusion

This study highlights how CHS nurses perceive challenges through their sense of professional responsibility in addressing childhood overweight, which seems to intensify when encountering parents with overweight. Furthermore, CHS nurses striving to fulfil this responsibility not only adopt a measurable and weight‐focused approach to health but may also question and distrust parents in ways shaped by the prevailing weight stigma in Western society. This appears to inform their approach during clinical encounters, in which they try to influence the parent to achieve a healthier weight for the child.

Future research should explore what guides CHS nurses in encounters with parents and children with overweight, how they construct their caring professional role in these situations and how they navigate—whether by resisting or reinforcing—the stigma surrounding overweight.

## Author Contributions

Conceptualisation and methodology: Julia Lindblom, Elzana Odzakovic, Eric A. Hodges, Maria Björk. Formal analysis: Julia Lindblom, Elzana Odzakovic, Eric A. Hodges, Anne Christenson, Maria Björk. Investigation and writing – original draft: Julia Lindblom. Supervision: Elzana Odzakovic, Eric A. Hodges, Anne Christenson, Maria Björk. Writing – review and editing: Julia Lindblom, Elzana Odzakovic, Eric A. Hodges, Anne Christenson, Maria Björk.

## Funding

The authors received no specific funding for this work.

## Ethics Statement

The study was approved by the Research Ethics Committee in Sweden, Dnr 2022‐03911‐02.

## Consent

Written informed consent was obtained from all participants involved in the study.

## Conflicts of Interest

The authors declare no conflicts of interest.

## Data Availability

The datasets generated during and/or analysed during the current study are not publicly available due to the sensitivity of the data but are available from the corresponding author on reasonable request.
